# Serum Vitamin E Status in Patients With Type 2 Diabetes Mellitus Among Bangladeshi Population

**DOI:** 10.1155/ghe3/9923689

**Published:** 2025-06-18

**Authors:** Monjurul Islam Ripon, Kazi Milenur Rahman Prattay, Uttom Kumar, A. S. M. Monjur Al Hossain, Muhammad Asaduzzaman, B. M. Redwan Matin Zidan, Sreedam Chandra Das

**Affiliations:** ^1^Department of Clinical Pharmacy and Pharmacology, University of Dhaka, Dhaka 1000, Bangladesh; ^2^School of Pharmacy, BRAC University, Dhaka 1212, Bangladesh; ^3^Department of Pharmaceutical Technology, University of Dhaka, Dhaka 1000, Bangladesh; ^4^Department of Pharmacy, University of Dhaka, Dhaka 1000, Bangladesh

**Keywords:** aging, alpha-tocopherol, sex-related effects, type 2 diabetes mellitus, vitamin E

## Abstract

**Background:** Type 2 diabetes mellitus (T2DM) links to oxidative stress in both its origin and progression. Vitamin E has the potential to be a highly effective therapeutic intervention in fighting against T2DM as it protects cells against oxidative stress. While some interventional studies have explored the effect of vitamin E on T2DM, there is a lack of cross-sectional studies globally, and none to our knowledge on the Bangladeshi population. Consequently, it is worthwhile to investigate the serum vitamin E levels in Bangladeshi T2DM patients.

**Methods:** 94 T2DM patients and 30 healthy subjects were evaluated for their serum vitamin E concentration for a comparative cross-sectional study. Mean serum concentrations were compared between these two groups, as well as among different sex and age groups using independent sample *t*-test and one-way ANOVA, as appropriate.

**Results:** The serum vitamin E concentration was significantly lower in T2DM patients (mean ± standard deviation: 8.97 ± 2.99 μg/mL) than in healthy subjects (13.13 ± 2.70 μg/mL), *p* < 0.001. Additionally, male T2DM patients had significantly higher serum vitamin E levels compared to those in female patients (9.73 ± 3.02 μg/mL in males vs. 7.74 ± 2.53 μg/mL in females; *p*=0.001). The study showed a significant fall in serum vitamin E concentration with increasing age in T2DM patients (≤ 30 years: 12.7 ± 1.05 μg/mL vs. 31–50 years: 11.06 ± 2.65 μg/mL vs. 51–70 years: 8 ± 2.04 μg/mL vs. 71–90 years: 6.05 ± 0.78 μg/mL; *p* < 0.001).

**Conclusion:** Our findings suggest that lower serum vitamin E levels are significantly associated with T2DM, particularly among female and older patients, highlighting the potential relevance of antioxidant status in T2DM management.

## 1. Introduction

The incidence of diabetes mellitus (DM) has seen a substantial rise during the last two decades, with the number of individuals diagnosed growing from 30 million to 285 million [1]. According to the diabetes global federation, if the present trend continues, the number of individuals with DM is projected to reach 438 million by 2030. DM is a metabolic illness characterized by the presence of oxidative stress in its pathophysiological mechanisms leading to compromised pancreatic function and/or dysregulation of insulin inside the human body. Although the global prevalence of both type 1 DM (T1DM) and type 2 DM (T2DM) is on the rise, T2DM is developing at a somewhat faster pace compared to T1DM [2]. Possible factors contributing to this pattern include a rise in obesity rates, growing elderly population, and a decrease in physical activity due to industrialization [3]. In 2010, around 1.6 million individuals, accounting for almost 20% of the population, were diagnosed with DM for the first time. The prevalence of DM is higher in the elderly population [4]. Global projections indicate that the majority of individuals with DM will fall between the age range of 45–64 years by 2030 [5]. Nevertheless, the occurrence of this condition remains rather consistent throughout all age cohorts, regardless of sex.

Vitamins are essential for human health. Studies on vitamin E and other vitamin supplements have shown positive effects on hypertension, blood glucose level, and antioxidant status [6–10]. Vitamin E (also known as alpha-tocopherol), categorized as a fat-soluble vitamin, has the capacity to eliminate free radicals and acts as an antioxidant. By engaging in this process, it effectively counteracts a diverse array of oxidative free radicals and has potential advantages in combating age-related conditions, namely, those associated with reactive oxygen species (ROS) [11]. Vitamin E has been associated with several medical conditions, including DM. T1DM is defined by a deficiency of insulin-producing *β*-cells in the pancreas, whereas T2DM is characterized by insulin resistance in the tissues it affects, such as adipose and muscle. Elevated blood glucose level is a distinctive sign of DM. The formation of ROS is a crucial factor in the progression of DM and its associated outcomes [12]. The production of superoxide radicals by the electron transport chain in mitochondria is increased in the presence of an excessive amount of intracellular glucose [13]. The antioxidant property of vitamin E is likely responsible for its ability to decrease the probability of developing DM and its associated problems [14, 15].

Oxidative stress has been shown to be associated with the onset and progression of DM, but the existing data are limited and often inconsistent. While there have been some interventional studies assessing the impact of vitamin E on T2DM, there is a notable gap in cross-sectional studies specifically assessing serum vitamin E levels in T2DM patients, particularly within the Bangladeshi population. This study aims to fill this gap by evaluating the serum vitamin E levels in individuals with T2DM in Bangladesh.

## 2. Materials and Methods

Current study was designed and conducted as a as a cross-sectional study based on biochemical assay of vitamin E levels to investigate the possible association between serum vitamin E level and presence of T2DM. The study also investigated if any statistically significant relationships between the patient's age, sex, and serum vitamin E levels exist in T2DM patients as well as in healthy volunteers.

### 2.1. Subject and Sampling

The research was conducted on a sample of 94 people diagnosed with T2DM, together with 30 individuals serving as healthy subjects (control group). A priori power analysis was conducted using G∗Power (Version 3.1.9.7), which indicated that two groups consisting of 94 patients (as the study group) and 30 healthy volunteers (as the control group) with an allocation ratio of 3:1 would be required to detect a medium effect size (*d* = 0.6) with 80% power at a 5% significance level. Our final sample size exceeds this requirement, ensuring sufficient power to detect meaningful differences. The participants were recruited using a convenience sampling method based on their availability and willingness to participate at the study site. Selected participants (both patients and healthy volunteers) underwent a screening to assess their blood glucose levels in fasting condition using a conventional glucometer. The study included T2DM patients with blood glucose levels found above 126 mg/dL in fasting condition and who were under no insulin therapy but oral medications. T1DM patients were carefully excluded from the study. Participants were included in the control group only if they had fasting blood glucose levels below 100 mg/dL and confirming that they were not taking any glucose-lowering medications immediately before and during the time of the study. All the participants from both the diseased and control groups had BMI values below 25 kg/m^2^ and were free of any diagnosed chronic inflammatory, cardiovascular, and chronic renal diseases [6].

### 2.2. Collection of Blood Sample

Five milliliters of venous blood samples was collected from each study participants from the Bangladesh Specialized Hospital (BSH), 21 Shyamoli, Mirpur Road, Dhaka-1207, Bangladesh, by following the standard laboratory protocol.

### 2.3. Preparation of Reagents for Analysis

#### 2.3.1. Standard Stock of Alpha-Tocopherol (0.27%, w/v)

A total of 270 mg alpha-tocopherol acetate was diluted first in 100 mL of absolute ethanol. Subsequently, the mixture was meticulously mixed.

#### 2.3.2. 2,2′-Bipyridyl (0.12%, w/v)

A solution of 120 mg of 2,2′-bipyridyl was prepared by dissolving it in n-propanol and raising the volume to 100 mL. Afterward, the mixture was meticulously mixed. The solution was then stored in an amber colored container until further use.

#### 2.3.3. Ferric Chloride, FeCl_3_ (0.12%, w/v)

A total of 120 mg equivalent of FeCl_3_.6H_2_O was dissolved in 100 mL of absolute ethanol and was preserved in an amber colored container until further use.

### 2.4. Preparation of Standard Curve

**Figure 1:**
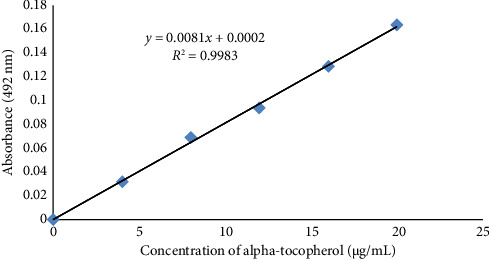
Standard curve of alpha-tocopherol (vitamin E) at 492 nm.

A 27 μg/mL alpha-tocopherol solution in absolute ethanol was prepared as a working standard solution using the standard stock solution of alpha-tocopherol (0.27%, w/v). Five different concentrations of alpha-tocopherol (4, 8, 12, 14, 16, and 20 μg/mL) were prepared from the working standard solution (27 μg/mL) adding the required amount of absolute ethanol to make the final volume 750 μL of each. From each of the prepared solutions, 200 μL was transferred onto the uncoated 96-well microplate and the absorbance was then measured at a wavelength of 492 nm using a multi-well plate reader. A standard curve was constructed by plotting the absorbances against the concentrations of alpha-tocopherol concentration (Figure 1).

### 2.5. Analysis of Alpha-Tocopherol in Serum

Blood samples collected in a centrifuge tube were subjected to centrifugation for 15 min at 3000 rpm. Subsequently, the blood serum was collected for further analysis. The method to determine the concentration of alpha-tocopherol was adapted from Jarger et al. [16]. While direct validation in the Bangladeshi population is limited, the method was initially reported upon investigation on Indian population which is very close to Bangladeshi population in terms dietary and lifestyle profiles as well physiological attributes. To ensure reliability, internal quality controls and standard reference materials were used during all assay procedures. Briefly, the sample tubes had been filled with absolute ethanol (750 μL) and serum (750 μL). To get a very distinct protein precipitate, the collected serum was gradually added to the tubes with continuous gentle mixing. A blank solution was prepared by adding 750 μL of distilled water to 750 μL of ethanol. The tubes were firmly wrapped with a wrapping paper and subjected to vigorous shaking for a minimum duration of 30 s. Subsequently, 750 μL of xylene was added into each of these tubes and vigorously shaken for another 30 s with wrapping, followed by centrifugation at 3000 rpm for 10 min.

The supernatant, consisting of 500 μL of xylene layer, was carefully transferred into appropriately labeled and sterile test tube. Each tube was treated with 500 μL of 2,2′-bipyridyl solution, followed by 100 μL of ferric chloride solution. The mixture was left undisturbed for 2 min. A 200 μL solution from each of these tubes was transferred to a 96-well clear microplate. All samples, including the blank, were analyzed for absorbance using a primary wavelength of 492 nm. Using the standard curve, the serum alpha-tocopherol concentration of each sample was determined. The serum concentration of each sample was the mean of the two independent assayed values.

### 2.6. Statistical Analysis

The data were analyzed using independent sample *t*-tests and one-way ANOVA, as appropriate, at a significance level of 0.05 to compare the mean serum vitamin E levels among different groups of participants. Normality was assessed using the Shapiro–Wilk test, and homogeneity of variances was tested using Levene's test. Independent sample *t*-tests were used to compare mean serum vitamin E levels between male and female participants. One-way ANOVA was used to compare the mean serum vitamin E levels across different age groups. Effect sizes (Hedges' *g* for *t*-tests and *η*^2^ for ANOVA) were also calculated to assess the practical significance of the findings. All statistical analyses were conducted using SPSS Statistics v26.0 except for the effect size (Hedges' *g*) of *t*-tests which was calculated using “Effect size calculator” of Social Science Statistics. All the graphs were constructed using GraphPad Prism v8.0.

## 3. Results

### 3.1. Demography of the Study Participants

A total of 124 individuals (94 patients and 30 healthy volunteers) participated in the study, comprising 74 males (59.68%) and 50 females (40.32%). The majority of participants were between 31 and 70 years of age, with 34.68% aged 31–50 years and another 34.68% aged 51–70 years. Participants aged ≤ 30 years accounted for 8.06%, while those aged 71–90 years represented 22.58% of the cohort. Regarding disease status, 94 participants (75.81%) were diagnosed with T2DM, and 30 (24.19%) were healthy subjects (Table 1).

### 3.2. Comparison of Serum Vitamin E Levels Between Participants With and Without T2DM

**Figure 2:**
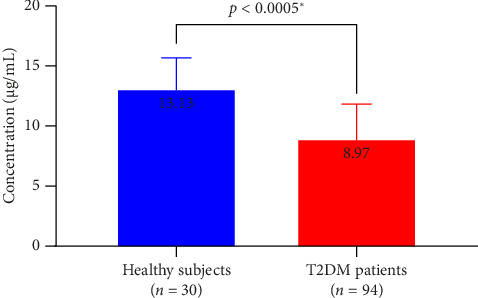
Serum vitamin E concentration in healthy subjects and patients with T2DM; the error bars represent the SD relative to the mean.

The mean serum concentrations of vitamin E among the participants with and without T2DM were 8.97 ± 2.99 μg/mL (mean ± standard deviation (SD)) and 13.13 ± 2.70 μg/mL, respectively, indicating a significantly lower serum vitamin E level in the T2DM patients (*p* < 0.001, Hedges' *g* = 1.42) (Figure 2).

### 3.3. Comparison of Serum Vitamin E Levels Between Male and Female Participants

Out of the 94 T2DM patients enrolled in this study, the number of male participants was 58 (61.70%) and the rest (38.30%) were female participants. The mean serum concentrations of vitamin E in male and female T2DM patients were estimated to be 9.73 ± 3.02 μg/mL and 7.74 ± 2.53 μg/mL, respectively. Notably, the serum level of vitamin E in male T2DM patients is significantly higher than that in female patients (*p*=0.001, Hedges' *g* = 0.70).

**Figure 3:**
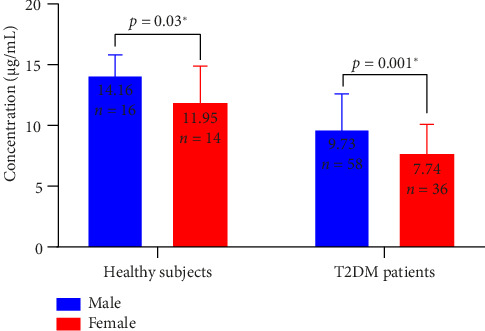
Comparison of serum vitamin E levels between sex in healthy subjects and T2DM patients; error bars represent standard deviation; the error bars represent the SD relative to the mean.

Furthermore, mean serum vitamin E levels in both healthy male and female participants (16 males and 14 females) were also determined and showed a pattern of finding which is similar to that of the T2DM patients. Although elevated in both male and female healthy individuals compared to T2DM patients, the mean serum concentration of vitamin E in male healthy subjects (14.16 ± 1.81 μg/mL) is significantly higher than that in female ones (11.95 ± 3.11 μg/mL) (*p*=0.03, Hedges' *g* = 0.88) (Figure 3).

### 3.4. Comparison of Serum Vitamin E Levels Across Age Groups

**Figure 4:**
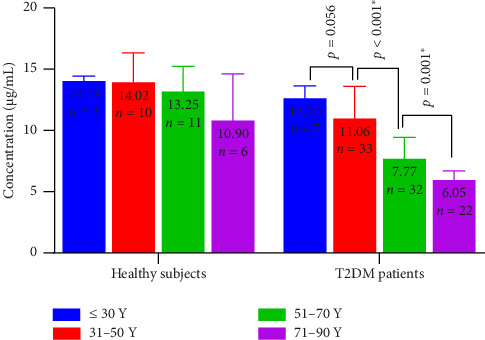
Comparison of serum vitamin E levels across different age groups in healthy volunteers and patients with T2DM. Error bars represent standard deviations relative to the mean; Y = years.

The findings of this study on serum vitamin E levels in individuals with T2DM indicate that there is a difference in the serum vitamin E level within age groups (Figure 4). In this study, the youngest group (age ≤ 30 years) had a mean serum vitamin E concentration of 12.7 ± 1.05 μg/mL, while the age group 31–50 years had 11.06 ± 2.65 μg/mL. Similarly, a downward trend in serum vitamin E level was also noted with the progressive age groups, as the patients with age groups 51–70 and 71–90 years showed the serum vitamin E levels 8 ± 2.04 μg/mL and 6.05 ± 0.78 μg/mL, respectively. One-way ANOVA study found a significant association between patient's age and the serum vitamin E level (*F* = 36.77, *p* < 0.001, *η*^2^ = 0.183). The post hoc analysis by the least significant difference (LSD) method confirmed that mean serum vitamin E level significantly decreased with increasing age of the patients (*p* < 0.001), with an exception between age groups ≤ 30 and 31–50 where the difference was not statistically significant (*p*=0.056) probably due to fewer number of samples with ≤ 30 age. As T2DM is mostly prevalent in older age people, patients with ≤ 30 age are rare.

In case of healthy volunteers, the mean serum vitamin E concentrations were found to be 14.13 ± 0.43 μg/mL, 14.02 ± 2.41 μg/mL, 13.25 ± 2.09 μg/mL, and 10.90 ± 3.85 μg/mL for participants belonging to age group of ≤ 30, 31–50, 51–70, and 71–90 years, respectively. Interestingly, although the serum vitamin E concentration showed a decline with age in healthy volunteers as well (Figure 4), it was not found statistically significant (*F* = 2.083, *p*=0.127, *η*^2^ = 0.19).

## 4. Discussion

The typical serum vitamin E concentration ranges from 5.7 to 19.9 μg/mL [17]. This study revealed that the average serum vitamin E concentration among the 94 individuals diagnosed with T2DM was 8.97 μg/mL, a value that falls within the acceptable range. The mean serum concentration of vitamin E in healthy volunteers was found to be 13.13 μg/mL, which is close to the findings of Bashar and Mitra [18].

Analysis of potential association between the serum vitamin E levels and T2DM in Bangladeshi population indicted that serum vitamin E level was found to be significantly lower in T2DM patients compared to that in healthy volunteers. These findings are consistent with multiple previous studies that opined a probable relationship between vitamin E and DM [19]. The study conducted by Salonen et al. included patient follow-up and tests of vitamin E levels and revealed that a deficit in vitamin E was associated with a 3.9-fold increase in the chance of developing DM. A reduction in vitamin E serum concentration by around 1 μM/L was predicted to elevate the risk of DM by approximately 22% when allowing for other strongest risk factors as well as serum low density lipoprotein cholesterol and triglyceride concentrations [20]. In another study, it was also revealed that the levels of vitamin E and vitamin C in persons with DM were considerably lower compared to those in the control group [21]. Such findings as explained by several research reports clearly suggest that the free radicals and lipid peroxidation play an obvious role in the development of oxidative stress and therefore in the etiology of DM. Pancreatic islet cells may exhibit increased susceptibility to damage from free radicals due to their comparatively lower levels of antioxidative enzyme activity [22, 23]. Vitamin E acts as an antioxidant and effectively eliminates a diverse array of oxidative free radicals from the body and, therefore, the high level of ROS in DM patients might result in greater consumption of vitamin E in this free radical scavenging process, consequently resulting in a low serum vitamin E level in the DM patients [12].

Nonetheless, a number of studies reported no significant association of vitamin E level and DM as well. The study conducted by Esteghamati et al. found that there were no significant changes in the levels of vitamin E and vitamin C in the plasma of DM patients compared to the control group, despite a drop in the ratio of vitamin E to cholesterol (*p* < 0.05) [24]. In a study conducted by Srivatsan et al., it was observed that the control group exhibited a vitamin E level of 1.08 mg/dL, while the diabetic patients with and without side effects showed vitamin E levels of 1.19 mg/dL and 1.17 mg/dL, respectively. No significant differences were found in vitamin E levels between the control group and the studied group [25]. Interestingly, Ble-Castillo et al. found that DM patients have greater amounts of vitamin E compared to healthy persons and that the elevated levels were deemed a protective factor [26].

Veglia et al. reported a strong association of age and sex in healthy volunteers with the overall oxidative status and with the concentration of specific antioxidants [27]. Therefore, these two variables might act as significant confounders in the comparison of serum vitamin E level in DM patients with healthy controls. Contradictory results reported in the literature may be explained by the failure to adjust for age and sex of the participants in the two study groups [27]. The current study investigated the relationship of sex and age with the serum vitamin E level in the diseased and control group separately. The mean serum vitamin E level in the female participants was found to be significantly lower than that in the male participants in both diseased and control groups. Multiple previous studies also reported a lower serum vitamin E level in female Asian population compared to male which supports our finding. The lower vitamin E levels observed in females may be biologically plausible due to several hormonal and metabolic mechanisms. Estrogen, the predominant female sex hormone, exerts antioxidant effects by upregulating the expression of antioxidant enzymes such as superoxide dismutase and glutathione peroxidase. This enhanced endogenous antioxidant defense may reduce the physiological demand for exogenous antioxidants like vitamin E, thereby influencing serum levels. In addition, estrogen can modulate lipid metabolism, impacting the absorption and transport of fat-soluble vitamins. Higher adipose tissue content in females may also contribute to increased sequestration of vitamin E in fat stores, resulting in lower circulating concentrations. Together, these factors support the biological plausibility of sex-specific differences in serum vitamin E levels [28–30]. We report here a significant decrease in serum vitamin E level with increasing the age in T2DM patients, although such decrease was not significant in the healthy individuals. Multiple studies reported a rise in the serum vitamin E levels with age among the healthy adults which is in contrast to our findings; however, a downfall in healthy elderly was reported in some other previous studies which again meets our report [30, 31]. The significant fall of vitamin E concentration with age in T2DM patients may be explained by the elevated consumption of vitamin E to counteract the increasing ROS activity in the aging patients [27].

However, this study has several limitations which include the small sample size, especially that of the control group, which may limit the generalizability of the findings. Additionally, dietary intake data were not collected, which restricts our ability to account for nutritional factors that may confound the observed associations. The cross-sectional design also limits the ability to infer causality between the variables studied. It demands future longitudinal investigation on a much larger sample size to ascertain a much robust association between the aforementioned parameters.

## 5. Conclusion

It is apparent from this study that the serum vitamin E level is significantly lower in patients with T2DM compared to the healthy subjects. Furthermore, the level of serum vitamin E is significantly lower in female than male and decreases gradually with age in the T2DM patients. However, for better comprehension, it is important to investigate on a larger cohort to retrieve further information and evidence on such association of low serum vitamin E level with T2DM. Given the observed association between low serum vitamin E levels and T2DM, further interventional research is warranted to evaluate whether vitamin E supplementation could play an important role in diabetes management and prevention at the clinical level.

## Figures and Tables

**Table 1:** Demography of the study participants.

	Frequency	Percentage (%)
Sex		
Male	74	59.68
Female	50	40.32
Age		
≤ 30	10	8.06
31–50	43	34.68
51–70	43	34.68
71–90	28	22.58
Disease status		
Healthy subject	30	24.19
T2DM patient	94	75.81

Abbreviation: T2DM = type 2 diabetes mellitus.

## Data Availability

The data that support the findings of this study are available from the corresponding author upon reasonable request.
